# Leptin’s crucial modulatory role in regulating body mass homeostasis of high-fat-fed striped field mice (*Apodemus agrarius*)

**DOI:** 10.3389/fphys.2025.1592317

**Published:** 2025-08-18

**Authors:** Yue Ren, Guangtong Guo, Yu Hou, Kuiyou Chen, Xinsheng Pu, Mengfan Tao, Yunlong Ren, Xin’gen Yang

**Affiliations:** ^1^ College of Plant Protection, Shanxi Agricultural University, Taiyuan, China; ^2^ Shanxi Key Laboratory of Integrated Pest Management in Agriculture, College of Plant Protection, Shanxi Agricultural University, Taiyuan, China

**Keywords:** *Apodemus agrarius*, leptin, body mass, high-fat food, AMPK

## Abstract

To investigate into the role of leptin in body mass in high-fat-fed animals. Male striped field mice (*Apodemus agrarius*) fed high-fat diets were given leptin (0.5 μg/g.d) via intraperitoneal injection for 28 days. Their body mass, digestive metrics, and physiological parameters of food consumption and energy metabolism were compared to those of the control and high-fat food groups. Firstly, the high-fat diet did not cause weight gain in *Apodemus agrarius*, and the animals on the diet ate less and had higher apparent digestibility. Furthermore, exogenous leptin injection in *A. agrarius* reduced food intake, increased fecal content, and reduced apparent digestibility. Additionally, exogenous leptin injection inhibited the activity of the AMPK in the hypothalamus, increased the activity of malonyl CoA, inhibited the expression of orexigenic neuropeptide mRNA, promoted the expression of anorexigenic neuropeptide mRNA, and thus reduced food intake and body mass. Finally, exogenous leptin injection increased uncoupling protein 1 content, T45′-deiodinase II activity, and cytochrome C oxidase activity in brown adipose tissue, increased serum triiodothyronine, and increased animal energy consumption. In conclusion, our data indicate that leptin affects body mass in animals on a high-fat diet in two ways: by inhibiting food intake and increasing energy expenditure.

## Introduction

Small animals typically respond to seasonal changes in their environment by altering a variety of physiological traits, including thermogenesis, food intake, body weight, and fat content (Heldmaier et al., 1982; Li and Wang, 2005). Central neural circuits and peripheral target tissues regulate appetite and energy balance in a coordinated and cohesive manner that includes negative feedback. The production and release of peripheral metabolic hormones from adipose tissue, such as leptin, is a crucial part of this negative feedback mechanism. Leptin is a 16-kD protein hormone with 167 amino acids. In humans and mammals, leptin is primarily synthesized and secreted by fat cells in white adipose tissue (Zhang et al., 1994). Leptin, a hormone signal for obesity, affects the central nervous system and helps mammals maintain energy homeostasis (Xing et al., 2016). It is critical for regulating animal food intake, body mass, and energy expenditure (Campfield et al., 1995; Pelleymounter et al., 1995; Halaas et al., 1997). Animals lacking leptin can become severely obese owing to two factors: increased food intake (Fischer et al., 2020; Nedergaard et al., 2023) and decreased energy production (Commins et al., 1999). Leptin primarily regulates food intake and energy expenditure via the hypothalamic receptor (Schwartz et al., 2000). High levels of leptin inhibit adenosine-5′-monophosphate-activated protein kinase (AMPK) activity in the hypothalamic arcuate nucleus (ARC) and paraventricular nucleus (PVN), preventing animal feeding (Minokoshi et al., 2004). Leptin is a stimulator of the AMPK pathway, leading to a downstream activation of this pathway, increasing malonyl CoA and inhibiting food intake (Wolfgang et al., 2007). Furthermore, numerous studies have found that leptin can increase the levels of anorectic peptides such as pro-opiomelanocortin (POMC) and cocaine- and amphetamine-regulated transcript (CART) mRNA while decreasing the levels of orexigenic peptides such as neuropeptide Y (NPY) and agouti gene-related protein (AgRP) mRNA (Flier, 2004; Morton et al., 2006). Leptin can also help the body lose weight by increasing energy expenditure. Exogenous leptin injections, for example, have been shown to stimulate sympathetic nerve activity in animals. This stimulation results in increased uncoupling protein 1 (UCP1) mRNA expression in brown adipose tissue (BAT), reduces body weight (Cancello et al., 1998; Demas et al., 2002; Gullicksen et al., 2002).

Leptin secretion is regulated by white adipose tissue (WAT) (Rousseau et al., 2003; Li and Wang, 2005), This has been found in the striped hairy-footed hamster (*Phodopus sungorus*), the ringnecked lemming (*Dicrostonyx groenlandicus*), the long-clucked gerbil (*Meriones unguiculatus*), and the great velvet mouse (*Eothenomys*) (Klingenspor et al., 2000; Johnson et al., 2004; Zhang and Wang, 2007; Zhu et al., 2010). So, leptin can be used as an indicator of energy availability (Nedergaard et al., 2023). The studies discovered that in small seasonal mammals such as Brandt’s vole (*Lasiopodomys brandtii*), *Dicrostonyx groenlandicus* and *Phodopus sungorus*, seasonal changes in food intake, body mass and body fat content were associated with seasonal changes in leptin levels (Klingenspor et al., 2000; Li and Wang, 2005). Exogenous leptin injection regulates energy metabolism homeostasis differently in different animals. Leptin injection, for example, can reduce food intake while increasing energy consumption in both ob/ob and wild-type mice (Pelleymounter et al., 1995; Levin et al., 1996; Johnson et al., 2004). In mice, both peripheral and central leptin injections reduced food intake and body fat (Halaas et al., 1997). During leptin injection under long light conditions, narrow-headed voles (*Microtus agrestis*) and *Lasiopodomys brandtii* showed leptin antagonism, but their food intake did not change (Klingenspor et al., 2000; Król and Speakman, 2007; Tang et al., 2009). Leptin injection can reduce food intake and heat production in F344×BN obese old rats (Shek and Scarpace, 2000). Wistar rats were injected with leptin at low temperatures, and their food intake, heat production capacity of BAT, and UCP1 concentration were all reduced (Abelenda et al., 2003). These findings suggest that leptin regulates energy homeostasis differently depending on the species. Furthermore, several studies have shown that other hormones, such as leptin, thyroid hormone, adiponectin, and ghrelin interact to regulate energy homeostasis and lipid metabolism (Zimmermann-Belsing et al., 2003; Hermann et al., 2006; Buonfiglio et al., 2018).

Striped field mice (*Apodemus agrarius*) are plant-eating, non-hibernating small mammals from the Muridae family and the genus *Apodemus*. It is widely distributed, plentiful, cold-resistant. According to previous research on the number and distribution of this mouse in Shanxi Province, this mouse has become one of the most common pests in typical farmland areas of Shanxi Province between 2015 and 2020 (Yang et al., 2021). It is also the primary carrier of epidemic hemorrhagic fever and leptospirosis (Zhang et al., 1997). This mouse’s reproduction (Wang et al., 1994), ecological habits (Wang et al., 1997), fatness, morphology (Yang, 1995; Yang, 2023), cold acclimation, and seasonal acclimation (Sun et al., 2009) were all extensively studied in the early period. However, researchers have not investigated the effect of exogenous leptin on body mass of *Apodemus agrarius*. This paper selected *A. agrarius* from Shanxi Province as the research object, and studied the effects of exogenous leptin on the feeding and energy metabolism of *A. agrarius* at different levels of individual, tissue, organs and biochemistry. It is predicted that exogenous leptin injection can reduce body weight by reducing food intake and enhancing heat production.

## Materials and methods

### Subjects and experimental design

Male striped field mice used in this study were captured from Xinzhou, Shanxi of China (113.3°E, 38.5°N) in September 2022. Animals were transported to and housed in Shanxi Agricultural University’s laboratory. The animals were housed individually in plastic cages (33 cm × 21.5 cm × 16 cm). Cages were kept at 24 °C ± 2 °C temperature and natural light cycle. Water and food (commercial rat chow; Shenyang QianMin Feed Co.) were provided *ad libitum*. All animals were kept in these conditions for at least 1 month before participating in the experiment. The animals were randomly divided into three groups based on body mass, including a control group, which was fed with abundant commercial rat chow (Con, *n* = 6); and a high energy food group, which was fed with abundant high-fat foods (HFD, *n* = 6); and a leptin injection group, which was both fed with abundant high-fat foods and injected with leptin intraperitoneal (HFD-leptin, *n* = 7), and the animals were injected with a PBS solution containing leptin at 50 ug per g of body mass (Li et al., 2020). The Con and HFD groups were injected with 1×PBS solution 50 ug per g of body mass (Chen et al., 2022). Table 1 shows the food composition. The experiment ran for 28 days. After 28 days, the animals were killed. The serum, medial hypothalamus, stomach, and retroperitoneal fat was collected and weighed, and then quickly frozen using liquid nitrogen until being stored at −80 °C. We also dissected and the remaining organs (Table 3). The animal’s body mass was measured every day (14:00–16:00). All animal operations follow the guidelines established by the Animal Care and Use Committee of Shanxi Agricultural University’s College of Veterinary Medicine. The committee approved this study.

**TABLE 1:** The food content.

Content	Standard diet	High-fat diet
Crude fat (%)	6.2	21.4
Crude protein (%)	20.8	17.6
Neutral detergent fiber (%)	21.5	19.6
Acid detergent fiber (%)	12.5	10.6
Ash (%)	10.0	8.5
Caloric value (kJ/g)	17.5	19.7

### Measurement of food consumption and digestibility

Food intake was measured using the food balance method (Liu et al., 2010). We provided food every 3 days. The amount of food missing from the hopper was calculated and dried in an oven at 60 °C. The average daily food intake was calculated by subtracting the dry weight of the missing food from the throw food. Faeces were collected every 3 days. The dry weight (±0.01 g) of the faeces was measured after drying in an oven at 60 °C. Daily faecal output (g/day/animal) = Total faecal dry weight/3; net food intake (g) = daily food intake - daily faecal output; and apparent digestibility is calculated as net food intake multiplied by 100% divided by daily food intake (Liu and Wang, 2007; Yin et al., 2019).

### Measurement of hormone concentration

Concentration of leptin in serum and white adipose tissue (WAT), as well as the concentration of adiponectin, T_3_, and T_4_ in serum were measured using ELISA kits (Preferred Biotechnology Co., Shanghai, China; Kit No: Leptin: JM-02902M1, Adiponectin: JM-02830M1, T_3_: JM-02857M2, T_4_: JM-02858M2), as described in previous studies (Ren et al., 2022; Liu et al., 2022).

### Measurement of leptin receptors, protein activity and neuropeptide mRNA expression in hypothalamic

The leptin receptors in hypothalamus were test using ELISA kits (Preferred Biotechnology Co., Shanghai, China; Kit No: Leptin receptor: JM-13102M2). AMPK activity and malonyl CoA activity in the hypothalamus were measured using enzyme-linked immunosorbent assay (ELISA) kits (Preferred Biotechnology Co., Shanghai, China; Kit No: AMPK: JM-03142M2, Malonyl CoA: JM-11403M2), as described in previous studied (Liu et al., 2022).

Total RNA was extracted from the hypothalamus utilising the TRIzol Kit (Invitrogen, Carlsbad, CA, United States), in accordance with the manufacturer’s guidelines (Liu et al., 2022). Isolated RNA was treated with DNase I (Promega, Madison, WI, United States of America) at 37 °C for 30 min to eliminate contaminating DNA, followed by an additional TRIzol extraction to eliminate residual DNase I. For each sample, 3 µg of total RNA was converted into first-strand cDNA utilising the M-MLV First Strand Kit (Invitrogen) according to the manufacturer’s guidelines (Preferred Biotechnology Co., Shanghai, China; Kit No: NPY: YX-E20502M; AGRP: YX-E22211M; POMC: YX-E22212M; CART: YX-E22217M). In accordance with the methodology outlined by Liu et al. (2022), cDNA was synthesised from total RNA. The real-time PCR was performed using the LightCycler System, a component of Roche Diagnostic GmbH’s (Mannheim, Germany) SYBR Green I sequence nonspecific detection method. Each PCR was conducted as previously reported (Liu et al., 2022). Table 2 outlines the cycling conditions and primers utilised; all primers were obtained from Sigma, Madrid, Spain. The software ABI7500 was used to read the Ct value of each PCR reaction. The Ct value of target gene subtract the value of reference gene as ^Δ^Ct, and ^Δ^Ct mean value of treatment group subtract ^Δ^Ct mean value of control group as ^ΔΔ^Ct, ans then the 2^−ΔΔCt^ represents the expression change of the treatment and control groups.

**TABLE 2:** Gene-specific primers used for real-time qPCR.

Primer	Oligonuncleotide sequence (5′–3′)	Product size (bp)
NPY (forward)	GTGTGGACTGACCCTCGCTCTATC	155
NPY (reverse)	TGGTGATGAGATTGATGTAGTGTCGC	
AgRP (forward)	GTGTTCTGCTGTTGGCACTG	157
AgRP (reverse)	ACTTCTTCTGCTCGGTCTGC	
POMC (forward)	CGCTGGAGACGCCCGTGTTTC	193
POMC (reverse)	CGTGGACTCGGCTCTGGACTGC	
CART (forward)	AGAAGAAGTACGGCCAAGTCC	84
CART (reverse)	CACACAGCTTCCCGATCC	
β-action (forward)	AGGTCATCACTATTGGCAACGAG	151
β-action (reverse)	TTGGCATAGAGGTCTTTACGGAT	

### Determination of protein content and enzyme activity in BAT

Rapidly dissect the animals, meticulously isolate the liver and interscapular BAT, weigh them to the nearest 0.001 g, and place them into 5 mL and 2 mL centrifuge tubes, respectively. Subsequently, immerse them in liquid nitrogen and transfer to a low-temperature freezer (−80 °C) for storage and preservation. Upon completion of sample collection, mitochondria from the liver and BAT were extracted using the Tissue Mitochondria Isolation Kit (Shanghai Biyuntian Biotechnology Co., Ltd., Product No. C3606). The UCP1 content, cytochrome C oxidase (COX, complex IV) activity, T_4_5′-deiodinaseII (T_4_5′-DII) activity in BAT were determined by mouse ELISA kits. UCP1 assay kit (Product No. JM-12185M2), COX assay kit (Product No. JM-11693M1), T_4_5′-DII assay kit (Product No. JM-13120M1) were purchased from Shanghai Preferred Biotechnology Co. (Shanghai, China). The experimental operation was carried out according to the instructions.

### Data analysis

Data were analysed using SPSS 26.0 software (SPSS Inc., Chicago, IL, United States). Before all statistical analyses, data were examined for normality and homogeneity of variance using Kolmogorov-Smirnov and Levene tests, respectively. Differences in organ masses among groups were examined using analysis of covariance (ANCOVA), with fat-free body mass as a covariate, followed by LSD *post hoc* tests. Continuous changes in body mass, food intake, daily faecal output, net food intake, and apparent digestibility were detected by repeated-measures ANOVA. Group disparities in food consumption, digestibility assessment, leptin and adiponectin levels, leptin receptor expression, and hypothalamic neuropeptide expression were evaluated using one-way analysis of variance (ANOVA) accompanied by LSD *post hoc* tests. The linear correlation among leptin content, hormone concentration, neuropeptide levels, and protein in BAT was examined using linear regression analysis. And relevant heat maps were created using online analysis software (https://www.genescloud.cn/hom). Results are expressed as means ± SEM, with *P* < 0.05 deemed statistically significant.

## Results

### Changes in body mass and organs mass

**FIGURE 1:**
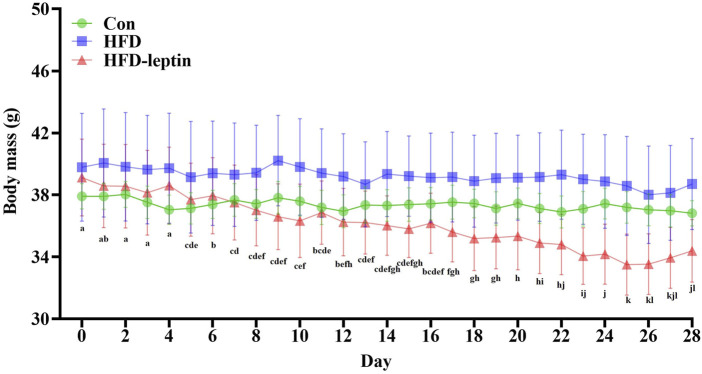
Changes in body mass of male striped field mice. Significant differences within group were indicated by different alphabetic letters. And significant between groups differences were indicated by *, and * as significant, *P* < 0.05, ** as extremely significant, *P* < 0.01. Significant differences in HFD-leptin group were indicated by different lowercase letter. Data are presented as mean ± SEM.

Over a 28-day monitoring period, the body masses of male striped field mice in the HFD-leptin group exhibited a significant reduction (*F* = 15.063, *P* = 0.01; Figure 1), with a decline of 4.734 g after 28 days. No significant difference was observed between the Con group and the HFD group (*P* > 0.05; Figure 1).

The organ mass results for the animals presented in Table 3 indicate that the stomach and cecum masses with food in the HFD group were significantly greater than those in the other two groups, whereas the wet weight of the stomach and cecum does not differ significantly among the three groups. Furthermore, the BAT mass in the HFD group was significantly lower than in the other groups, while the HFD-leptin group exhibited the highest BAT mass (*F* = 5.961, *P* < 0.05). The kidney mass in the HFD group and the HFD-leptin group was significantly greater than that in the Con group (*F* = 4.772, *P* < 0.05). The organ masses exhibited no significant differences among the three groups (*P* > 0.05).

**TABLE 3:** Group differences in organ masses of male striped field mice.

Organs mass	Con	HFD	HFD-leptin	*F*value	*P*value
Stomach with food (g)	0.500 ± 0.086^c^	1.742 ± 0.159^a^	1.060 ± 0.139^b^	21.271	<0.01
Stomach (g)	0.276 ± 0.029	0.360 ± 0.079	0.250 ± 0.042	1.051	>0.05
Liver (g)	0.930 ± 0.215	1.480 ± 0.152	1.961 ± 0.375	3.257	>0.05
Heart (g)	0.172 ± 0.017	0.241 ± 0.017	0.232 ± 0.032	2.437	>0.05
Lungs (g)	0.192 ± 0.015	0.236 ± 0.033	0.241 ± 0.026	0.982	>0.05
Kidney (g)	0.236 ± 0.027^b^	0.350 ± 0.037^a^	0.357 ± 0.029^a^	4.772	<0.05
Caecum with food (g)	0.527 ± 0.065^b^	0.372 ± 0.038^a^	0.362 ± 0.035^a^	5.364	<0.05
Caecum (g)	0.215 ± 0.012	0.232 ± 0.037	0.209 ± 0.028	0.166	>0.05
Small intestine (g)	0.654 ± 0.081	0.556 ± 0.052	0.591 ± 0.085	0.131	>0.05
Large intestine (g)	0.202 ± 0.021	0.233 ± 0.032	0.179 ± 0.023	1.017	>0.05
BAT (g)	0.183 ± 0.020^ab^	0.100 ± 0.064^b^	0.317 ± 0.033^a^	5.961	<0.05

Data were analyzed by one-way ANOVA, followed by the LSD, *post hoc*test. Significant group differences were indicated by different alphabetic letters. Data are presented as mean ± SEM.

### Food consumption and digestibility

**FIGURE 2:**
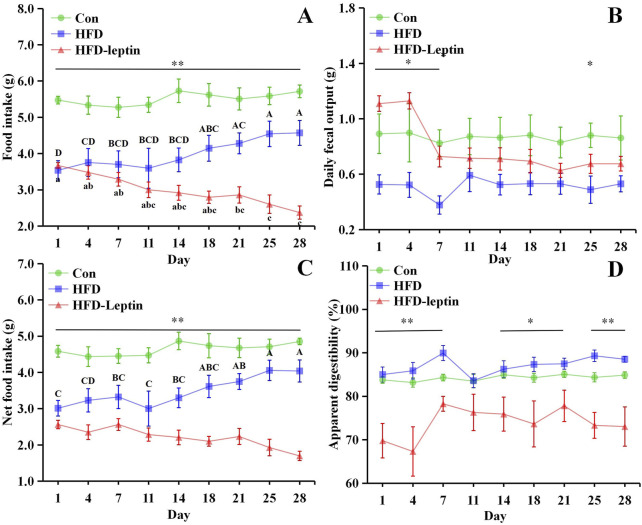
Effect of leptin on food intake **(A)**, daily fecal output **(B)**, net food intake **(C)**, and apparent digestibility **(D)**. Significant differences within group were indicated by different alphabetic letters. And significant between groups differences were indicated by *, and * as significant, *P* < 0.05, ** as extremely significant, *P* < 0.01. Significant differences in HFD and HFD-Leptin group were indicated by different capital letter and lowercase letter, respectively. Data are presented as mean ± SEM.

The variations in food intake, daily fecal output, net food intake, and apparent digestibility were shown in Figure 2. Firstly, the Con group exhibited a significantly higher food intake than the other groups over the 28-day period (*P* < 0.05; Figure 2A). After 14 days, the HFD group commenced consuming significantly greater quantities of food than the HFD-leptin group (*F* = 23.690, *P* < 0.05). Food intake of HFD group significantly increased from 18 days (*F* = 4.654, *P* < 0.01). Furthermore, the food intake of the HFD-leptin group exhibited a significant reduction starting from day 14 (*F* = 32.737, *P* < 0.01). Secondly, a noteworthy discovery indicated a disparity between the alterations in food consumption and the modifications in faecal dry weight. The HFD-leptin group demonstrated a significantly reduced food intake compared to the HFD group, but additionally exhibiting a notably increased faecal dry weight (*P* < 0.05, Figure 2B). The faecal dry weight of the HFD-leptin group was significantly greater than that of the Con group on both day one and day four (P < 0.05). Furthermore, the faecal dry weight of the high-fat diet was the lowest. Moreover, akin to the food consumption outcomes, the net intake of the Con group was markedly greater than that of the other groups (*P* < 0.01, Figure 2C). After 14 days, the net intake of the HFD group was significantly greater than that of the HFD-leptin group (*P* < 0.05, Figure 2C). The HFD group exhibited greater apparent digestibility compared to the Con group. The HFD-leptin group exhibited significantly lower apparent digestibility compared to both the HFD group and the Con group (*P* < 0.01). Our data indicate that the HFD-leptin group exhibited the least food intake, while exhibiting a significant faecal dry weight, leading to the lowest apparent digestibility (Figures 2A,B,D).

### Measurement of hormone concentration in serum

**FIGURE 3:**
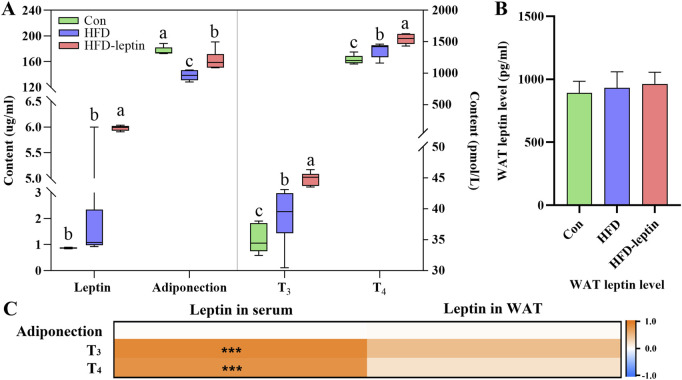
Effect of leptin on serum hormone levels in *Apodemus agrarius* fed high fat diet. The changes of serum hormone **(A)**. The change of leptin on WAT **(B)**, and correlation analysis between leptin and serum hormone **(C)**. Adiponectin Significant group differences were indicated by different alphabetic letters, *P* < 0.05.

**FIGURE 4:**
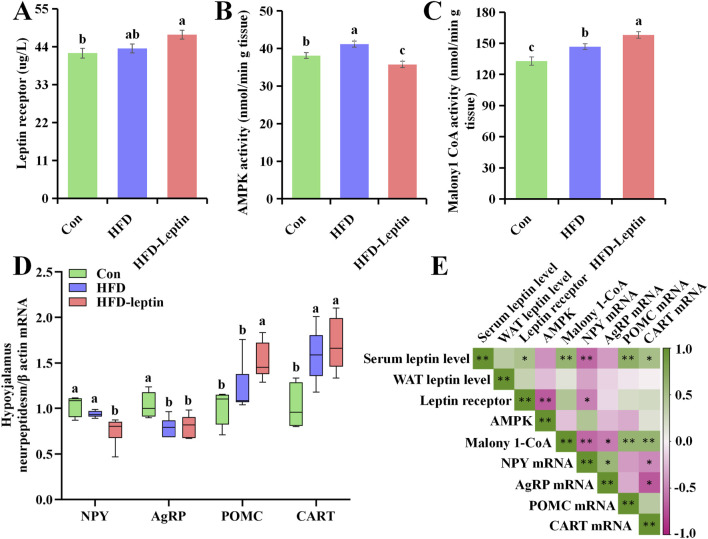
Leptin receptor **(A)**, activity of AMPK **(B)**, malonyl CoA **(C)**, as well as expression of hypothalamus neuropeptidesm mRNA **(D)** in *Apodemus agrarius*, and correlation heat map **(E)**. Significant group differences were indicated by different alphabetic letters, *P* < 0.05.

The levels of leptin, adiponection, T_3_, and T_4_ in the serum were shown in Figure 4. The serum levels of leptin T_3_, and T_4_ in serum exhibited similar fluctuated among three groups. First, the HFD-leptin had a significant higher level of leptin in serum than the other groups (*F* = 32.206, *P* < 0.01, Figure 3A). However, there was no significant difference in WAT leptin levels among the three groups (*F* = 0.617, *P* > 0.05). Additionally, HFD-leptin subjects exhibited elevated levels of T3 and T4 compared to the control subjects (*F*_*T3*_ = 18.034, *P* < 0.01; *F*_*T4*_ = 16.466, *P* < 0.01; Figure 3A). Furthermore, the serum T_3_ and T_4_ levels in the HFD group were higher than those in the Con group (*P* < 0.05). Adiponectin levels in the Con group were noticeably higher than those of the other groups; the HFD-leptin group had higher levels than the HFD group (*F*_adiponection_ = 20.390, *P* < 0.01). Furthermore, WAT leptin level did not vary among the three animal groups (*F* = 0.746, *P* > 0.05, Figure 3B). At last, the results of the hormone correlation analysis revealed that serum leptin level had a significant correlation with T3 in serum (*R* = 0.769, *P* < 0.01) and T4 in serum (*R* = 0.833, *P* > 0.05), but not with adiponectin in serum (*R* = 0.020, *P* > 0.05). WAT leptin level had not been recorded (*R*_adiponection_ = 0.296, *R*_*T3*_ = 0.209, *R*_*T4*_ = 0.400, *P* > 0.05, Figure 3C).

### Expression of leptin receptor, and AMPK activity, and malonyl CoA activity in hypothalamusm, and hypothalamus neuropeptidesm

The expression of the leptin receptor in the hypothalamus varied among animals from different groups (*F* = 4.253, *P* < 0.05, Figure 4A). Compared to the HFD and Con animals, the HFD-leptin animals demonstrated an increased quantity of leptin receptors in the hypothalamus. The AMPK activity in the HFD group significantly increased compared to the other groups (*F* = 11.047, *P* < 0.01, Figure 4B). The HFD-leptin group exhibited the lowest AMPK activity. Ultimately, a notable disparity in malonyl CoA activity was observed among the three groups (*F* = 14.345, *P* < 0.01, Figure 4C). The HFD-leptin group exhibited the highest malonyl CoA activity, followed by the HFD group, with the Con group exhibiting the lowest activity.

**FIGURE 5:**
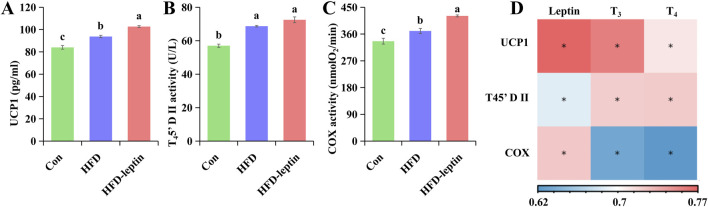
Effect of leptin on protein content and enzyme activity in BAT. Leptin affects UCP1 content **(A)**, T_4_5′-DII activity **(B)** and COX activity **(C)** in BAT of *Apodemus agrarius*. And correlation analysis between serum hormone and protein content as well as enzyme activity in BAT **(D)**. Significant group differences were indicated by different alphabetic letters, *P* < 0.05.

The results of four hypothalamus neuropeptidesm mRNA in hypoyhalamus, including NPY, AgRP, POMC, and CART were shown in Figure 5E. There was significant difference between three groups (Figure 5E). First, the expression of NPY mRNA of HFD-leptin group significantly lower than the other groups (*F* = 9.984, *P* < 0.01). While there was no difference between Con and HFD group (*P* > 0.05). Second, the expression of AgRP mRNA of HFD group and HFD-leptin group was both significantly lower than that of Con group (*F* = 8.327, *P* < 0.01). There was no difference between HFD group and HFD-leptin group (*P* > 0.05). Moreover, the expression of POMC mRNA of HFD-leptin group significantly higher than that of the other groups (*F* = 9.289, *P* < 0.01). There was no difference between HFD group and HFD-leptin group (*P* > 0.05). Finally, the expression of CART mRNA of Con group was significantly lower than that of the other groups, and there was no difference between HFD group and HFD-leptin group (*F* = 10.478, *P* < 0.01).

We further analyzed the correlation between leptin content, leptin receptor with hypothalamic protein activity and hypothalamus neuropeptides (Figure 4E). The findings indicated that serum leptin levels exhibited a significant positive correlation with leptin receptor (*R* = 0.491, *P* < 0.05), malonyl CoA activity (*R* = 0.636, *P* < 0.01), and the expression of POMC mRNA (*R* = 0.680, *P* < 0.01) and CART mRNA (*R* = 0.504, *P* < 0.05), while demonstrating a negative correlation with the expression of NPY mRNA (*R* = 0.621, *P* < 0.01). No correlation was observed between leptin content and WAT leptin levels (*R* = 0.382, *P* > 0.05), AMPK activity (*R* = 0.435, *P* > 0.05), as well as AgRP mRNA expression (*R* = 0.399, *P* > 0.05). Furthermore, no correlation was observed between WAT leptin levels and leptin receptor, AMPK activity, malonyl CoA activity in the hypothalamus, or hypothalamic neuropeptides (*R*_*leptin receptor*_ = 0.317, *R*_*AMPK*_ activity = 0.229, *R*_*malonyl CoA*_ = 0.127, *R*_*NPY*_ = 0.352, *R*_*AgRP*_ = 0.136, *R*_*POMC*_ = 0.042, *R*_*CART*_ = 0.012, *P* > 0.05). Additionally, a negative correlation was observed between leptin receptor levels in the hypothalamus and AMPK activity (*R* = 0.680, *P* < 0.01), as well as the expression of NPY mRNA (*R* = 516, *P* < 0.05). There was no correlation between leptin receptor activity in the hypothalamus and malonyl CoA activity, as well as the expression levels of AgRP, POMC, and CART mRNA (*R*_*malonyl CoA activity*_ = 0.458, *R*_*AgRP*_ = 0.111, *R*_*POMC*_ = 0.210, *R*_*CART*_ = 0.225, *P* > 0.05). AMPK activity exhibits no correlation with malonyl CoA activity or hypothalamic neuropeptides (*R*_*malonyl CoA activity*_ = 0.218, *R*_*NPY*_ = 0.456, *R*_*AgRP*_ = 0.242, *R*_*POMC*_ = 0.310, *R*_*CART*_ = 0.125, *P* > 0.05). The results indicated a significant correlation between malonyl CoA activity and hypothalamic neuropeptides, exhibiting a positive correlation with POMC mRNA (*R* = 0.628, *P* < 0.01) and CART mRNA (*R* = 0.639, *P* < 0.01), and a negative correlation with NPY mRNA (*R* = 0.633, *P* < 0.01) and AgRP mRNA (*R* = 0.527, *P* < 0.05).

### Protein content and enzyme activity in BAT

The results of protein content and enzyme activity in BAT were shown in Figure 5. The content of UCP1 and COX activity in BAT of the HFD-leptin group was significantly higher than in other groups (*F*_*UCP1*_ = 57.168, *P* < 0.01; *F*_*COX*_ = 27.571, *P* < 0.01, Figures 5A,B). Moreover, the T_4_5′-DII activity of the HFD-leptin group and the HFD group was significantly higher than the Con group (*F*_*T45'-DII*_ = 48.301, *P* < 0.01, Figure 2B). In addition, the content of UCP1 and COX activity of the HFD group was significantly higher than that of the Con group.

Moreover, the result showed that there were significant correlations between serum leptin and UCP1(*R* = 0.773, *P* < 0.01), as well as the activity of COX (*R* = 0.773, *P* < 0.01) and T_4_5′-DII (*R* = 0.773, *P* < 0.01) in BAT, and there were significant correlations between serum T_3_ and T_4_ as well as UCP1 (*R*_*T3*_ = 0.760, *R*_*T4*_ = 0.710, *P* < 0.01), as well as the activity of COX (*R*_*T3*_ = 0.635, *R*_*T4*_ = 0.622, *P* < 0.01) and T_4_5′-DII (*R*_*T3*_ = 0.721, *R*_*T4*_ = 0.723, *P* < 0.01) in BAT (Figure 5D).

## Discussion

The adaptation of animals to their external environment will be directly reflected in their body mass (Li and Wang, 2005). Preserving a consistent body mass is essential for the survival and reproduction of small mammals. Various factors affect animal body mass, with food quality serving as a crucial determinant. For instance, the body mass and adipose tissue levels of experimental rats and mice consuming buffet-style or high-fat diets increased markedly (Rothwell and Stock, 1998). Nevertheless, high-fat diets did not result in body mass increase in certain wild rodent species, including *P. sungorus* prairie vole (*Microtus pennsylvanicus*) (El-Bakry et al., 1999), *L. brandtii* (Zhao et al., 2008), Chevrier’s field mouse (*Apodemus chevrieri*) (Gao et al., 2013a), striped hamster (*Cricetulus barabensis*) (Bi et al., 2018), and red-backed vole (*Eothenomys miletus*) (Geng et al., 2024). In this study, following 28 days of high-fat diet administration, the body mass of *A. agrarius* exhibited no significant change, paralleling observations in other rodent species. The quality of food is the primary determinant influencing the energy intake and digestibility of animals, subsequently impacting the equilibrium of energy metabolism. Research indicates that high-fat diets can diminish food intake, energy intake, and digestible energy in *Apodemus chevrieri* (Gao et al., 2013a), *L. brandtii* (Zhao et al., 2008), and *Cricetulus barabensis* (Bi et al., 2018), while markedly enhancing their digestibility. In the current study, analogous results were noted. Consumption of high-fat food diminished the food intake of *A. agrarius*, consequently decreasing its fecal output and enhancing its apparent digestibility. In contrast to the findings of acclimation with high-fiber diets in Ryukyu mice (*Mus Caroli*, Bonhote, 1902), high-fiber diets markedly elevated the daily food consumption and fecal output of the mice, while the apparent digestibility significantly declined (Yin et al., 2019). Voltura and Wunder (1998) posited that when confronted with varying food quality, rodents typically augmented their food consumption to adjust to low-quality foods and offset low digestibility. Enhancing the digestibility and optimizing the utilization efficiency of food enables adaptation to high-quality nutrition (Voltura and Wunder, 1998).

Leptin is a crucial protein hormone that regulates body mass in animals (Friedman and Halaas, 1998; Ahrén et al., 1997; Abelenda et al., 2003; Tang et al., 2009; Chen et al., 2022; Picó et al., 2022). This study demonstrated that exogenous leptin injection diminished the body mass of animals on a high-fat diet, corroborating findings from other studies involving leptin-injected animals (Li et al., 2020). Leptin primarily functions in various regions of the central nervous system and peripheral tissues to modulate body mass, encompassing two facets: diminishing food consumption and enhancing energy expenditure (Halaas et al., 1995; Pelleymounter et al., 1995; Levin et al., 1996; Friedman and Halaas, 1998; Concannon et al., 2001; Johnson et al., 2004; Teixeira et al., 2021). Leptin administration can diminish food consumption and enhance energy expenditure in ob/ob mice, ultimately resulting in substantial weight reduction (Halaas et al., 1995; Pelleymounter et al., 1995; Levin et al., 1996). Additional small mammals, including the black field mouse (*Micratus agrestis*) (Król and Speakman, 2006), *P. sungorus* (Klingenspor et al., 2000), *E. miletus* (Chen et al., 2022; Chen et al., 2023), and rats (Abelenda et al., 2003), exhibited analogous outcomes when administered exogenous leptin. The findings of this study indicated that the exogenous administration of leptin diminished the food consumption of *A. agrarius*, paralleling results from other rodent studies. The daily fecal output of animals administered leptin increased, while its apparent digestibility significantly decreased, potentially elucidating leptin’s role in body weight regulation. Leptin decreases food consumption in animals while simultaneously increasing fecal output, diminishing digestibility, lowering food utilization efficiency, and consequently reducing animal weight. The mechanism by which leptin regulates digestibility requires further investigation. The gut microbiota is a crucial element in the regulation of food digestion in animals (Clarke et al., 2014). The exogenous administration of leptin may influence the digestive function of animals by altering the composition and structure of gut microbiota (Li et al., 2020), which will be further investigated by our research team subsequently.

Modifications to the morphology of the digestive tract are intricately associated with the energy requirements of an organism. Enabling animals to regulate the weight and shape of their digestive tracts is essential for animal empowerment (Wang et al., 1995; Wang et al., 2009; Gao et al., 2013b). When the external environment alters, numerous small mammals can acclimate to variations in food quality by modifying their digestive systems, including increasing the food turnover rate and altering the digestive tract volume (Gross et al., 1985; Bozinovic et al., 1990; Sassi et al., 2007). Numerous studies have examined the digestive tracts of small mammals, revealing that the trends in digestive tract variation differ among species and under varying conditions (Wang and Wang, 2000). The morphology of the digestive tract in animals is influenced by variations in food quality, which subsequently affects their digestive capacity and efficiency (Karasov, 1996). The total digestive tract contents of *Apodemus alpine*, when fed a high-fat diet, exhibited a significant reduction (Gao et al., 2013b). Nonetheless, the mass of the stomach and large intestine of *A. alpine* subjected to a diet rich in sugar and fat exhibited a significant increase (Yang et al., 2024). This study identified phenotypic alterations in the mass of certain internal organs. These morphological alterations may signify modified functions (Wang et al., 2003). The results indicate that the stomach weight of *A. agrarius*, when fed a high-fat diet, is significantly lower than that of the control group, potentially due to an increase in stomach volume in response to varying food quality (Chediack et al., 2012; Gao et al., 2013b). Nonetheless, the gastric weight of *A. agrarius* administered exogenous leptin and fed a high-fat diet was markedly reduced compared to both the high-fat group and the control group, potentially attributable to diminished food consumption, as the stomach serves as the organ for temporary food storage and initial digestion and absorption. Nonetheless, there was no variation in the weight of gastric contents among the three groups. Secondly, the findings of this study indicate that the cecal content in the standard food group is markedly greater than that in the other two groups, and the reactions of various small rodents to high-fat diets resemble those of *A. agrarius*; for instance, the cecum of *A. alpine* subjected to a high-fat diet was significantly smaller than that of the low-fat food group (Gao et al., 2013b). The cecum serves as the locus for cellulose fermentation, primarily indicating alterations in food quality. Elevated cellulose content in food leads to an increase in the cecum, as high-fiber foods are primarily fermented and digested there (Liu and Wang, 2007). Theoretically, if the volume of the digestive tract remains constant, the turnover rate of high-fat foods should decrease, resulting in prolonged food retention time and enhanced digestibility. *A. agrarius* can enhance absorption efficiency by decreasing the turnover rate of food to adapt to high-quality sustenance and sustain energy equilibrium. The kidneys of animals have experienced substantial alterations. These findings collectively provide further evidence that animals can demonstrate phenotypic plasticity in response to environmental changes by preserving essential traits and discarding non-essential ones (Caumul and Polly, 2005).

Leptin inhibits AMPK activity in the hypothalamic arcuate and paraventricular nuclei by influencing the hypothalamic receptor (obR), thereby suppressing feeding behavior in animals (Schwartz et al., 2000). Leptin simultaneously influences downstream substances of the AMPK pathway to elevate malonyl coenzyme A, diminish the secretion of orexigenic neuropeptides and enhance the release of anorexigenic neuropeptides, thereby suppressing food intake (Flier, 2004; Morton et al., 2006; Wolfgang et al., 2007). Our data indicate that plasma leptin can suppress AMPK activity in the hypothalamus and reduce food consumption (Stark et al., 2013; Liu et al., 2022). The present results indicate that the levels of leptin and leptin receptors in the hypothalamus of the HFD-leptin group are the highest, followed by the HFD group, while AMPK activity in the hypothalamus of the HFD-leptin group is significantly lower than in the other two groups. Furthermore, our data indicate that leptin can directly elevate malonyl-CoA to suppress food consumption (Stark et al., 2013; Wolfgang et al., 2007). Previous research on *A. agrarius* indicated that malonyl-CoA activity was maximal in the HFD-leptin group, and significantly elevated in the HFD group compared to the Con group, potentially elucidating the reduced food intake observed in the HFD group. The findings of this study indicated that the expression of NPY mRNA and AgRP mRNA in the hypothalamus of the HFD group was diminished compared to the control group, whereas the expression of POMC mRNA and CART mRNA was elevated relative to the control group. This may result in reduced food consumption in the HFD group compared to the Con group (Flier, 2004; Morton et al., 2006). Administering exogenous leptin to animals consuming a high-fat diet augmented the suppression of NPY mRNA and AgRP mRNA expression while enhancing POMC mRNA and CART mRNA expression in the hypothalamus, resulting in a further decrease in food intake and ultimately facilitating weight loss in these animals. Collectively, these data provide more evidence suggesting that an increase in leptin levels not only inhibits the AMPK pathway but also increases the content of malonyl-CoA, thereby reducing the expression of orexigenic neuropeptides and increasing the expression of anorexigenic neuropeptide, which in turn leads to reduced food intake in animals and ultimately lowers their body mass (Flier, 2004; Morton et al., 2006; Wolfgang et al., 2007).

Leptin can not only affect body mass by inhibiting food intake but also regulate body mass by increasing energy consumption (Stark et al., 2013; Asgari et al., 2025). Leptin can enhance the uncoupling protein 1 (UCP1) levels in BAT to modulate thermogenic capacity, subsequently elevating the body’s energy expenditure (Scarpace et al., 1997; Scarpace and Matheny, 1998; Commins et al., 2001). Our data support the conclusion that leptin enhances the thermogenic activity of BAT. The findings indicated that the UCP1 levels in the BAT of the HFD-leptin group were the highest, succeeded by the HFD group, while the Con group exhibited the lowest UCP1 content. A high concentration of leptin enhances the thermogenic capacity of melatonin in BAT (Li and Wang, 2005; Chen et al., 2022; Chen et al., 2023). The elevated mass of BAT in the HFD-leptin group may suggest that elevated leptin levels can enhance BAT thermogenesis (Pelleymounter et al., 1995; Levin et al., 1996; Commins et al., 1999; Scarpace et al., 1997; Chen et al., 2022; Chen et al., 2023). Leptin deficiency results in reduced UCP1 expression and diminished thermogenic capacity in animals. Exogenous leptin supplementation can rectify this deficiency (Commins et al., 1999). In addition, compared with the Con group, the UCP1 of HFD group also increased significantly, which may be due to the fact that animals consume excess energy by increasing heat production when they consume too much energy, thus maintaining a constant body mass.

In addition to UCP1, the activities of T_4_5′-DII and COX are significant contributors to the enhanced thermogenesis in small mammals. T_4_5′-DII can locally convert T_4_ into active T_3_, which is the principal mechanism by which thyroid hormones regulate metabolism (Mullur et al., 2014). The research indicates that elevated serum T_3_ levels may account for the heightened deiodinase activity in animals administered leptin over an extended period (Cettour-Rose et al., 2002). Leptin can enhance the activity of T_4_5′-DII (Cettour-Rose et al., 2002; Lisboa et al., 2003). The capacity of the T_4_5′-DII gene to eliminate heat production in mouse BAT diminished (de Git et al., 2019). The application of T_4_5′-DII inhibitors may result in reduced UCP1 expression in BAT (Branco et al., 1999). In this study, serum leptin levels exhibit a positive correlation with T_4_5′-DII activity and sreum T_3_ and T_4_ levels, while T_4_5′-DII activity also shows a positive correlation with serum T_3_ and T_4_ levels. The data suggest that leptin enhances the activity of T_4_5′-DII, thereby facilitating the conversion of more T_4_ into T_3_, ultimately augmenting heat production and energy expenditure in animals (Chen et al., 2022). Ultimately, we discovered that COX activity in the BAT of the HFD-Leptin group increased significantly, suggesting that elevated leptin levels enhanced the overall respiratory capacity of BAT (Chen et al., 2022).

The experimental results in this study support the notion that exogenous leptin reduces the body mass of animals through two pathways: one is by reducing the animals’ food intake, and the other is by increasing their heat production. Similar results were also obtained in other small rodents. However, due to species differences and some objective factors, our experimental results differ slightly from those of other experiments. Nevertheless, the overall trend is consistent.

## Conclusion

In this study, we investigated the effect of exogenous leptin injection on body mass regulation of high-fat-fed *A. agrarius*. First, it is interesting to note that the high-fat diet did not cause weight gain in *A. agrarius*, and the animals on the high-fat diet ate less and increased their apparent digestibility. Moreover, compared to high-fat diet animals, peripheral injection of leptin in *A. agrarius* further limited their food intake, increased fecal content, and decreased their apparent digestibility. However, the reasons why leptin increases the amount of animal feces and the mechanism of its action on animal digestibility are still unclear. In addition, peripheral leptin injection inhibits the activity of AMPK in the hypothalamus, increases the activity of malonyl-CoA, and then inhibits the expression of orexigenic neuropeptide mRNA, promotes the expression of anorexigenic neuropeptide mRNA, and then reduces food intake and reduces body weight. Last but not least, exogenous leptin injection increased UCP1 protein content, T_4_5′-DII activity, and COX activity in BAT, increased serum T_3_, and increased animal energy consumption. In conclusion, our data illustrate that leptin affects body mass in animals on a high-fat diet in two ways, including inhibiting food intake and increasing energy expenditure.

## Data Availability

The datasets presented in this study can be found in online repositories. The names of the repository/repositories and accession number(s) can be found below: Physiological data are available supporting this study on Figshare at https://doi.org/10.6084/m9.figshare.28396460.v1.
